# Case of Extra-Uterine Pregnancy

**Published:** 1848-05

**Authors:** Samuel A. Peters

**Affiliations:** Boone county, Missouri; Philadelphia


*Case of Extra Uterine Pregnancy.* By Saml. A. Peters, M. D., of Boone county, Missouri.

On the 17th of December 1847, I was summoned to see a mulatto woman, the property of Mr. ———, in Boone county, Mo., who was taken very suddenly ill. Not being at home when called for, I did not get to see the patient until an hour and a half had elapsed from the time of her being taken ill.

*History.*—This woman, æt. 35, formerly enjoyed tolerably good health; had seven children, and was supposed to be pregnant at this time; this, however, had not been satisfactorily ascertained. She stated, that three months anterior to her present illness, her menstrua had not appeared at the regular time, but since then, they were twice visible at irregular intervals. The owner of the woman informed me on my arrival, that she had been washing the floor of a room, not having complained of being unwell, when, suddenly, she fell down. She was placed upon a bed, and they supposed her complaint to be nothing more than a "fainting spell," which they said she had had several times before. Her pulse was ninety-six, and barely perceptible; tongue natural; lips pallid; extremities extremely cold. She referred "her misery" to the hypogastric region. I pressed my hand upon this part of the abdomen, but this neither lessened nor increased "her misery." I examined *per vaginam*, but could discover nothing that would throw any light upon the nature of the case. The lips of the os uteri were thick and soft, and I could easily introduce one of my fingers into it.

From not being able to satisfy myself positively, from the phenomena that presented themselves, as to the nature of the case, or the cause of such a sudden enervation of the system, I was necessitated to prescribe for symptoms. I supposed, after making use of stimulants, linaments, fomentations, &c., from the patient still becoming weaker, with all the concomitant symptoms, that there certainly must be internal hemorrhage. She died two hours from the commencement of the attack.

*Post mortem examination.* The cavity of the abdomen was filled with a large quantity of fluid blood. I removed *fifteen pints* before I could advance further with the examination. An embryo with its membranes, was partly adhering to the upper anterior part of the bladder. The chorion, and amnion, and placenta, were distinctly seen. The embryo is three inches in length; all the external members are perfectly formed; it had one coil of the funis around its neck. I removed the uterus and its appendages; the former was three times its natural size, and three-fourths of an inch in thickness. The inner surface presented no appearance of a membrane lining it. Had a decidua been there, or any gelatinous or fibrinous matter been present, I should certainly have detected it. The glandulæ Nabothi were slightly enlarged. The Fallopian tubes presented nothing unnatural, externally, or internally. The orifices had the appearance, at the angles of the uterus, which they usually have in the unimpregnated state. The right ovarium was much larger than the left, and when cut into, I observed that one of the Graafian vesicles was enlarged, and filled with a brownish coloured fluid.

Judging from the size of the embryo, and also from what the patient said respecting her menses, I should suppose that she had been pregnant about three months.

The cause of the sudden death is obvious. The adhesions of the membranes of the ovum to the bladder becoming ruptured or broken,—the bloodvessels of the bladder being consequently opened, and not having the power to contract, they permitted the blood to flow from them until death ensued.

I was sorry, that it was impossible to have a medical witness present at this examination, to confirm me in the position that there was no deciduous membrane lining the cavity of the uterus. I am well aware, that the major part of writers on embryology, affirm, that the decidua is formed in the uterus before the ovum arrives there, or is commencing to be formed at the time the ovum is impregnated in the Graafian vesicle. This membrane I certainly was not able to discover in the above case of ventral pregnancy.

*Philadelphia, March 21st,* 1848.

